# Identification of the Pyroptosis-Related Gene Signature and Risk Score Model for Colon Adenocarcinoma

**DOI:** 10.3389/fgene.2021.771847

**Published:** 2021-12-06

**Authors:** Bixian Luo, Jianwei Lin, Wei Cai, Mingliang Wang

**Affiliations:** Department of General Surgery, Ruijin Hospital, Shanghai Jiao Tong University School of Medicine, Shanghai, China

**Keywords:** pyroptosis, colon adenocarcinoma, risk score, gene signature, nomogram

## Abstract

The prognosis of advanced colon adenocarcinoma (COAD) remains poor. However, existing methods are still difficult to assess patient prognosis. Pyroptosis, a lytic and inflammatory process of programmed cell death caused by the gasdermin protein, is involved in the development and progression of various tumors. Moreover, there are no related studies using pyroptosis-related genes to construct a model to predict the prognosis of COAD patients. Thus, in this study, bioinformatics methods were used to analyze the data of COAD patients downloaded from The Cancer Genome Atlas (TCGA) and Gene Expression Omnibus (GEO) databases to construct a risk model for the patient prognosis. TCGA database was used as the training set, and GSE39582 downloaded from GEO was used as the validation set. A total of 24 pyroptosis-related genes shown significantly different expression between normal and tumor tissues in COAD and seven genes (CASP4, CASP5, CASP9, IL6, NOD1, PJVK, and PRKACA) screened by univariate and LASSO cox regression analysis were used to construct the risk model. The receiver operating characteristic (ROC) and Kaplan–Meier (K–M curves) curves showed that the model based on pyroptosis-related genes can be used to predict the prognosis of COAD and can be validated by the external cohort well. Then, the clinicopathological factors were combined with the risk score to establish a nomogram with a C-index of 0.774. In addition, tissue validation results also showed that CASP4, CASP5, PRKACA, and NOD1 were differentially expressed between tumor and normal tissues from COAD patients. In conclusion, the risk model based on the pyroptosis-related gene can be used to assess the prognosis of COAD patients well, and the related genes may become the potential targets for treatment.

## Introduction

Colon cancer is one of the most common cancers in the world (Bray et al., 2018). Colon adenocarcinoma (COAD) is the most common type of colon cancer (Fleming et al., 2012). The main treatment for colorectal adenocarcinoma is surgery, but the 5-year survival of patients is not satisfactory due to postoperative recurrence and metastasis (Zhai et al., 2017). Biomarkers have been used to aid in identifying patients at high risk of tumor progression or recurrence, such as the RAS mutation state, BRAF mutation state, and microsatellite instability (MSI) state (Sepulveda et al., 2017; Koncina et al., 2020). Therefore, the determination of molecular changes in COAD patients has become a focus of COAD research.

Pyroptosis is a lytic and inflammatory process of programmed cell death caused by the gasdermin protein (Wang et al., 2017). The members of gasdermin families include GSDMA, GSDMB, GSDMC, GSDMD, GSDME (also known as DFNA5), and PJVK (also known as DFNB59) (Broz et al., 2020). In contrast to apoptosis, pyroptosis can cause plasma membrane rupture, pore formation, cytoplasmic swelling, and chromatin condensation (Fink and Cookson, 2006). After cell rupture, proinflammatory cytokines and immunogenic substances are released to promote immune cell activation and infiltration, which may result in a strong inflammatory response and significant tumor regression (Wang et al., 2017; Loveless et al., 2021). Increasing studies indicated that pyroptosis may play important roles in the development of many cancers (Al Mamun et al., 2021). In COAD, pyroptosis may participate in the tumorigenesis of cancer (Tan et al., 2020; Tang et al., 2020). Meanwhile, pyroptosis induction can increase the chemosensitivity of COAD (Guo et al., 2021). Hence, pyroptosis-related genes may become the potential biomarkers to predict the prognosis of COAD and provide guidance for treatment.

In this study, bioinformatics was used to determine the expression levels of relevant genes between normal tissues and tumor tissues in COAD to explore the prognostic value of these genes. Then, a risk model based on pyroptosis-related genes was constructed by univariate and LASSO cox regression analysis. Moreover, a nomogram established by the clinicopathological features and risk model was used to further improve the prognostic ability in COAD. Finally, the tumor tissue and paired normal tissue from 13 patients with COAD were used to verify the gene expression in the model.

## Methods

### Data Collection

The mRNA expression data (Workflow Type: HT seq-FPKM) and relevant clinical information for COAD patients downloaded from TCGA website (https://portal.gdc.cancer.gov/repository) on 2 August, 2021, were used as the training set. There are 437 samples (39 normal tissues and 398 COAD tissues) and 384 cases of COAD patients being collected. The data of GSE39582 from GEO (http://www.ncbi.nlm.nih.gov/geo/) was used as the validation set. A total of 579 cases of COAD patients were collected. The GEO samples were analyzed using the Affymetrix Human Genome U133 Plus 2.0 Array platform. Patients from TCGA and GEO with missing clinical information were deleted in subsequent studies. Clinicopathological characteristics of patients (age, gender, stage, T stage, N stage, and M stage) were recorded.

### Identification of Differentially Expressed Genes

The pyroptosis-related genes were obtained from the research about ovarian cancer (Ye et al., 2021). The expression data of these pyroptosis-related genes were downloaded from TCGA databases. The “limma” package of R language was regarded as the method to identify differentially expressed genes (DEGs) with the P-value <0.05. The significance of DEGs was marked as follows: * for P-value < 0.05, ** for P-value < 0.01, and *** for P-value <0.001. The STRING database (http://string-db.org/) was set for searching the online for possible interactions between related genes. The PPI network of DEGs was constructed by this database.

### Construction of the Risk Score Model by Univariate Cox and LASSO Cox Regression Analyses

Univariate regression analysis was used to screen pyroptosis-related genes associated with prognosis. The cutoff P-value was set to 0.2 to prevent omissions. LASSO analysis with the “glmnet” R package was utilized to construct the risk score model after univariate regression analysis. Each COAD patient risk score was calculated by this model and used to divide patients into two groups (low-risk and high-risk groups) by the median value. The receiver operating characteristic (ROC) and Kaplan–Meier (K–M curves) curves were used to evaluate the prognostic ability of the risk model. PCA was analyzed by R language with the “stats” package. GSE39582 was regarded as the validation set to verify the predictive ability of the prognostic risk model based on TCGA database. In addition, univariate and multivariate Cox regression analyses were also used for verifying whether the risk model was associated with prognosis and could be used as an independent prognostic risk factor for COAD.

### Functional Enrichment Analysis and Alteration of Seven Genes in the Model

The cBioPortal dataset (https://www.cbioportal.org/), which contains genomic data from 104 different tumors, was used to study genetic variations of the genes in our model. GeneMANIA (http://www.genemania.org)contains genetic information, analysis of gene lists, and functional analysis of prioritized genes, with high predictive algorithms (Warde-Farley et al., 2010). Thus, GeneMANIA was used to analyze genes interacting with gene models and their enrichment function.

### Construction of the Nomogram to Estimate the Clinical Outcome of COAD Patients

The “rms” R package was used to construct a nomogram. Calibration curves were used to test association between the predicted outcome and the actual situation in 1-, 3-, and 5 -year survival rate. GSE39582 was the validation dataset for the nomogram.

### Correlation of the Genes and Risk Score with Clinicopathological Features and Immune Cells as well as Immune Signaling Pathways

The “beeswarm” R package was used to assess the correlation of the genes and risk score with clinicopathological features. The tumor immune microenvironment is the key to tumor–anti-tumor immunity. The “gsva” R package was utilized to conduct the ssGSEA to calculate the scores of infiltrating immune cells and to evaluate the activity of immune-related pathways.

### Tissue Sample Collection

A total of thirteen pairs of fresh tumor tissues from COAD patients and their paired normal tissues were collected from Ruijin Hospital of Shanghai Jiao Tong University, Shanghai Jiao Tong University School of Medicine, which was approved by the Human Research Ethics Committee of this hospital. All fresh samples were stored at −80°C for the following experiments.

### Ethics Statement

This study was approved by the Human Ethics Committee of Ruijin Hospital. Informed consent was obtained from all enrolled patients and healthy donors.

### Quantitative Real‐Time PCR

The total RNAs from tissues were extracted with the Trizol reagent (Invitrogen, CA, USA). The NanoDrop 2000 spectrophotometer (Thermo) was used to quantify RNA, and cDNA was generated by the PrimeScript™ RT Reagent Kit (Takara, China) and then analyzed using RT-qPCR with the TB Green® Premix Ex Taq™ II (Takara, China) on the 7,500 Fast Real-Time PCR System (Applied Biosystems, CA, USA). β-actin was exploited as an internal reference. The mRNA relative expression of individual genes was detected by 2^−ΔCt^ methods. The primer sequences used for analysis are listed in Table 1.

**TABLE 1:** Primer sequences of genes in the risk model used for qPCR.

Gene	Primer sequence
β-actin	F:GACCTGTACGCCAACACAGT
	R:CTCAGGAGGAGCAATGATCT
CASP4	F:TCTGCGGAACTGTGCATGATG
	R:TGTGTGATGAAGATAGAGCCCAT
CASP5	F:TTCAACACCACATAACGTGTCC
	R:GTCAAGGTTGCTCGTTCTATGG
CASP9	F:CTGTCTACGGCACAGATGGAT
	R:GGGACTCGTCTTCAGGGGAA
IL6	F:ACTCACCTCTTCAGAACGAATTG
	R:CCATCTTTGGAAGGTTCAGGTTG
NOD1	F:ACTGAAAAGCAATCGGGAACTT
	R:CACACACAATCTCCGCATCTT
PJVK	F:CTGGATCAGATTCCATTGCAGT
	R:GTGGTTCGGATGCTCTCCAT
PRKACA	F:CAAGGAGACCGGGAACCACTA
	R:CATTCAGGGTGTGTTCGATCTG

qPCR, quantitative real-time polymerase chain reaction.

### Statistical Analysis

R version 4.0.5, Perl version 5.28, and Graphpad Prism 8.0.2.263 were used for statistical analysis. TCGA and GEO data were organized by Excel Office 2019. Except that the P-value < 0.2 was set as the condition for screening prognostic genes in univariate Cox regression analysis, the P-value < 0.05 was used as the significant condition for others without special explanation.

## Results

### Overall Design of the Study

**FIGURE 1:**
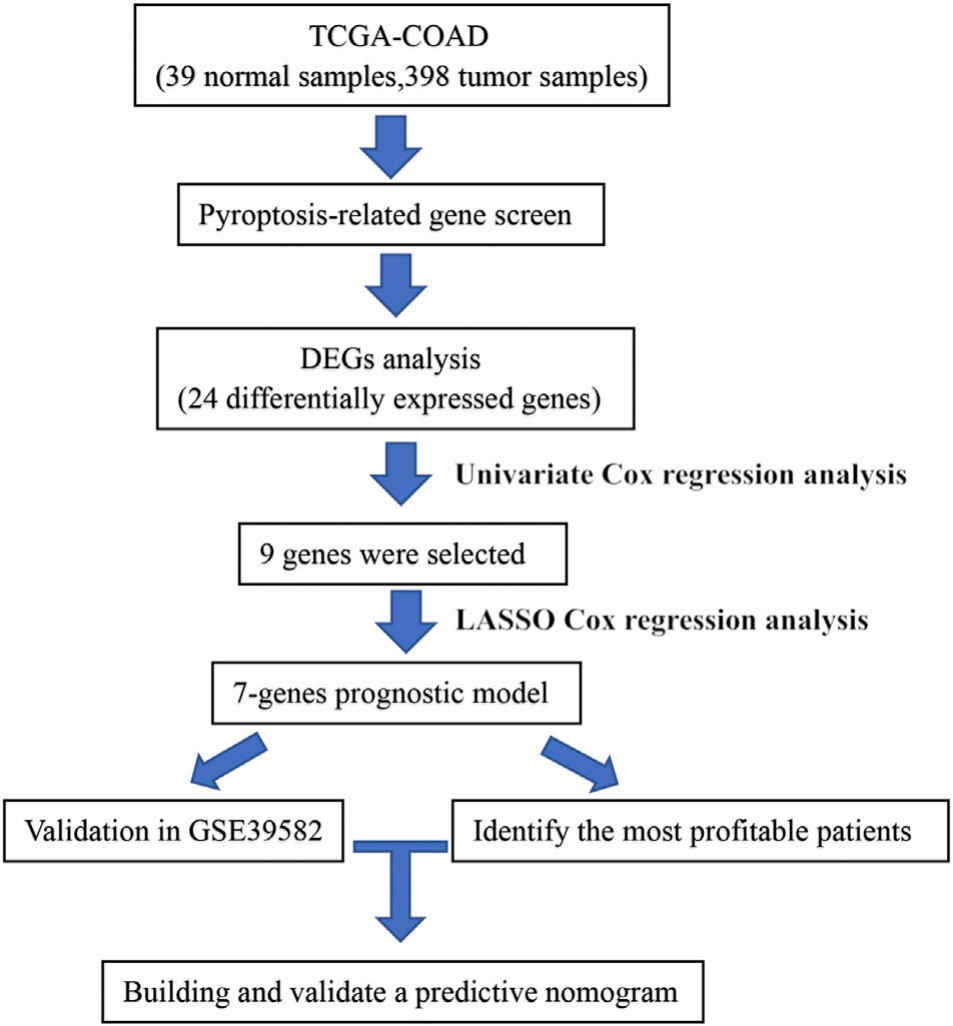
Workflow to construct the pyroptosis-related risk model in COAD patients. TCGA, The Cancer Genome Atlas; COAD, colon adenocarcinoma; DEG, differentially expressed gene.

The flow chart of this study is shown in Figure 1. The relevant clinical information of patients from TCGA and GEO is shown in Table2. There were 24 pyroptosis-related genes being screened. A total of seven genes were selected after univariate and LASSO Cox regression analysis. The ROC and K–M curves were used to evaluate the prognostic ability of the risk model based on the seven pyroptosis-related genes. The GSE39582 from GEO was used as the external cohort to validate the model and nomogram. The calibration and C-index verified the predictive ability of the nomogram.

**TABLE 2:** Clinical information of COAD patients from TCGA and GEO.

		TCGA	GEO
		n = 384	n = 579
Age	≤65	159	227
	>65	225	351
	Unknown	0	1
Gender	Female	180	260
	Male	204	319
Stage	Stage 0	0	4
	Stage I–II	216	306
	Stage III–IV	157	269
	Unknown	11	0
T	T0	0	1
	T1–2	77	60
	T3–4	306	495
	Tis	1	3
	Unknown	0	20
M	M0	285	496
	M1	54	61
	Mx	39	2
	Unknown	6	20
N	N0	230	311
	N1–3	154	242
	Unknown	0	26

T, T stage; N, N stage; M, M stage; Stage, TNM, stage.

### Identification of Differentially Expressed Genes

**FIGURE 2:**
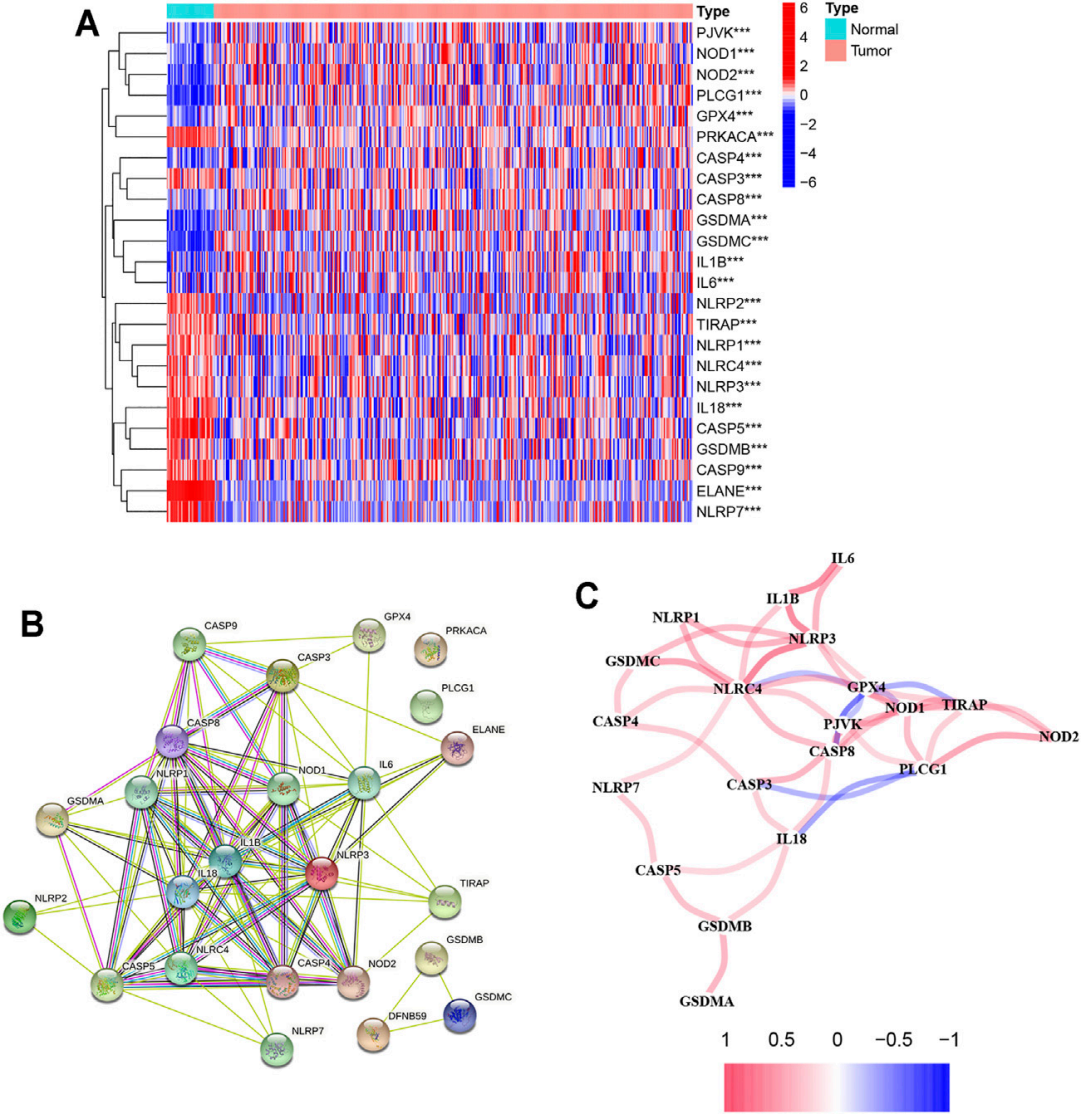
Results of differential gene analysis. **(A)** Heatmap of differentially expressed pyroptosis-related genes. The vertical axis refers to genes, the horizontal axis refers to differences in the gene expression between tissues, the orange means high expression, and the blue means low expression. P-values are shown as: **p < 0.01; ***p < 0.001; **(B)** PPI network showing the interactions of differentially expressed pyroptosis-related genes; **(C)** correlation of the differentially expressed pyroptosis-related genes (red line: positive correlation; blue line: negative correlation. The depth of the colors reflects the strength of the relevance).

There were 24 pyroptosis-related genes screened by analyzing the data from TCGA database which is shown in Figure 2A. Of these genes, 11 genes were upregulated (CASP8, NOD1, GPX4, CASP4, PJVK, IL6, IL1B, PLCG1, NOD2, GSDMA, and GSDMC) and 13 genes were downregulated (ELANE, CASP5, NLRP7, IL18, NLRP3, NLRC4, PRKACA, NLRP1, GSDMB, CASP9, CASP3, TIRAP, and NLRP2) between normal and tumor tissues. The PPI network based on the STRING database showed IL18, IL1B, IL6, NLRP3, NLRP1, NLRC4, CASP5, NOD1, CASP8, NOD2, CASP4, and CASP3 were the key genes which interacted with more other genes (Figure 2B). The correlation network containing 24 pyroptosis-related genes is shown in Figure 2C.

### Tumor Classification Based on the Differentially Expressed Pyroptosis-Related Genes

**FIGURE 3:**
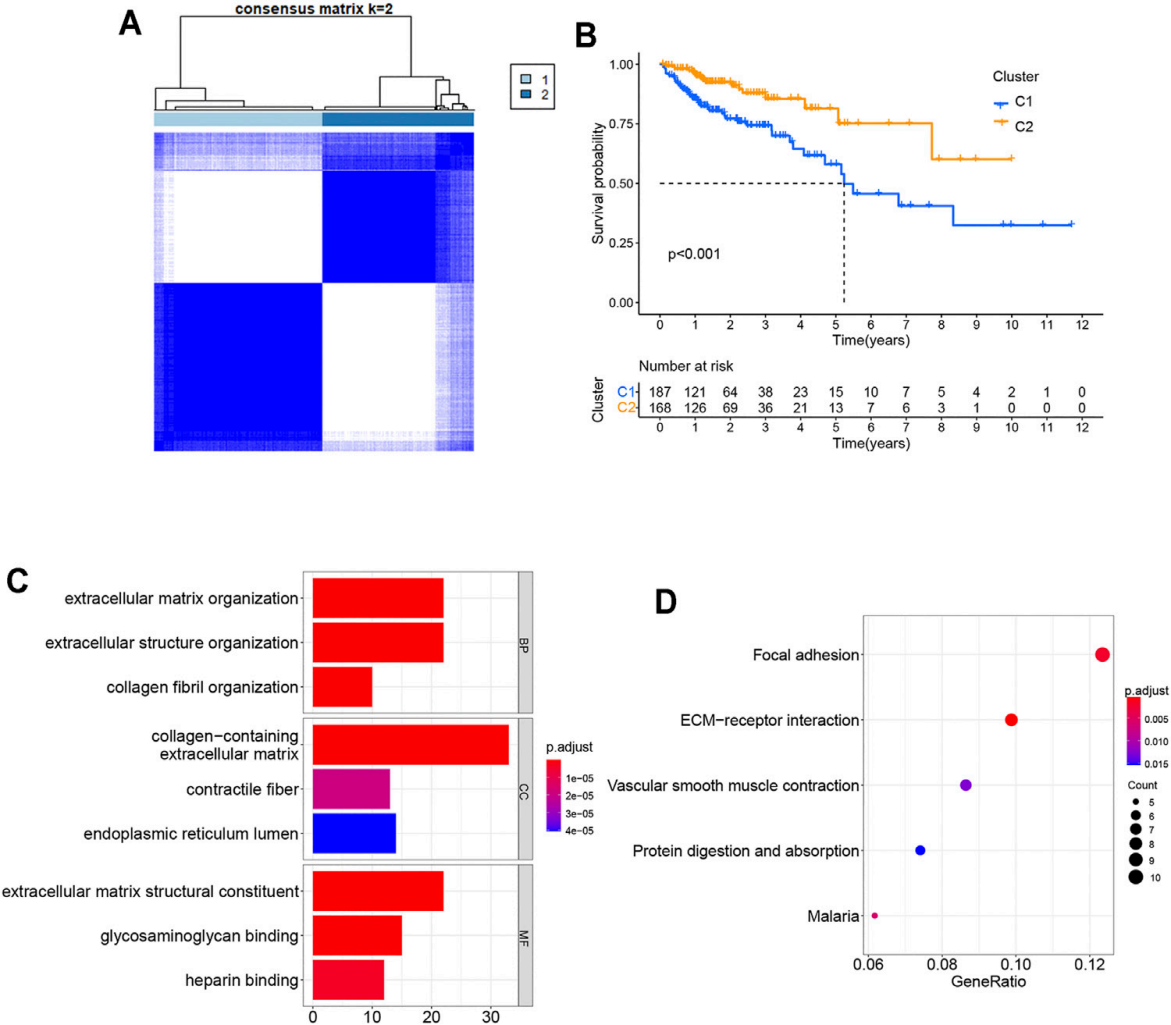
Tumor classification based on the pyroptosis-related DEGs. **(A)** COAD patients were grouped into two clusters according to the consensus clustering matrix (k = 2). **(B)** Kaplan–Meier OS curves for the two clusters. **(C)** GO bar graph for genes in BP, CC, and MF. **(D)** Bubble graph of top 5 KEGG pathways with the most enriched genes; the vertical axis refers to names of the pathway; and the horizontal axis refers to the number of genes. GO, Gene Ontology; BP, biological process; CC, cellular component; MF, molecular function; KEGG, Kyoto Encyclopedia of Genes and Genomes.

To explore the relationship between the 24 pyroptosis-related genes and COAD subtypes, the consensus clustering analysis was used to analyze the patients from TCGA database. The patients whose follow-up time was less than 30 days were excluded from the consensus clustering analysis. As the clustering variable (k) increased (from 2 to 10), intragroup connections were the highest and intergroup connections were the lowest when k = 2, indicating that the COAD patients could be well divided into two clusters based on the 24 differentially expressed genes (DEGs) (Figure 3A). The overall survival (OS) time was also compared between two clusters and showed great differences (Figure 3B). DEGs and clinical features between two groups are shown in Supplementary Figure S1, indicating that 207 genes and stages M stage and N stage were significantly different. Gene Ontology (GO) analysis for 207 genes showed that these genes participated in extracellular matrix organization, extracellular structure organization, collagen fibril organization, and so on (Figure 3C). The Kyoto Encyclopedia of Genes and Genomes (KEGG) pathway enrichment analysis for 207 genes is shown in Figure 3D.

### Risk Score Model

**FIGURE 4:**
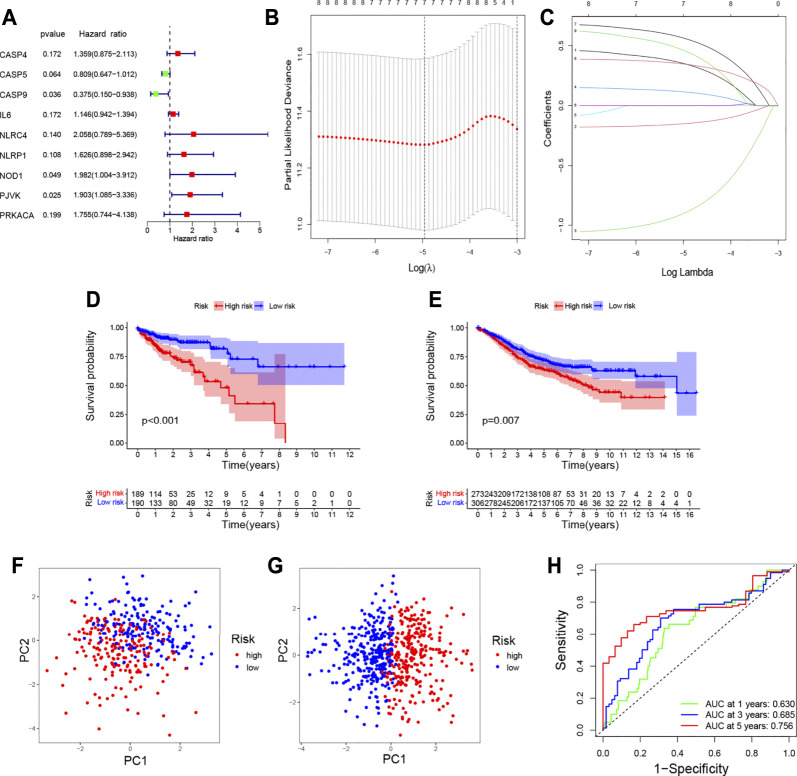
Construction of the prognostic model for COAD. **(A)** Hazard ratio of univariate Cox analysis for pyroptosis-related DEGs; **(B)** distribution of LASSO coefficients for seven genes. Two vertical lines represent lambda. min and lambda. lse; **(C)** coefficients for seven genes analyzed by LASSO; survival analysis to verify the prognostic model in TCGA **(D)** and GEO **(E)**; PCA plot for COAD based on the risk score in TCGA **(F)** and GEO **(G)**; **(H)** time-dependent ROC curves for COAD.

Univariate Cox regression analysis was performed on 24 differentially expressed pyroptosis-related genes in TCGA database with 384 COAD patients. When the P-value was set to 0.2, nine genes were identified (Figure 4A). Then, LASSO Cox regression analysis showed that seven genes could be used to construct prognostic risk models in the optimum λ value (Figures 4B,C). The risk score was calculated as follows:

risk score=(CASP4∗0.34668)+(CASP5∗−0.15059)+(CASP9∗−0.89296)+(IL6∗0.11284)+(NOD1∗0.55807)+(PJVK∗0.34451)+(PRKACA∗0.45435).

Based on the median risk score, the patients from TCGA and GEO databases were divided into two groups (low-risk and high-risk groups). As shown in Figure 4D, the prognosis was worse in the high-risk group. In addition, the validation set proved that the risk model based on pyroptosis-related genes could well predict the prognosis of patients (Figure 4E). The principal component analysis (PCA) showed that patients with different risks were well separated into two clusters (Figures 4F,G).

**FIGURE 5:**
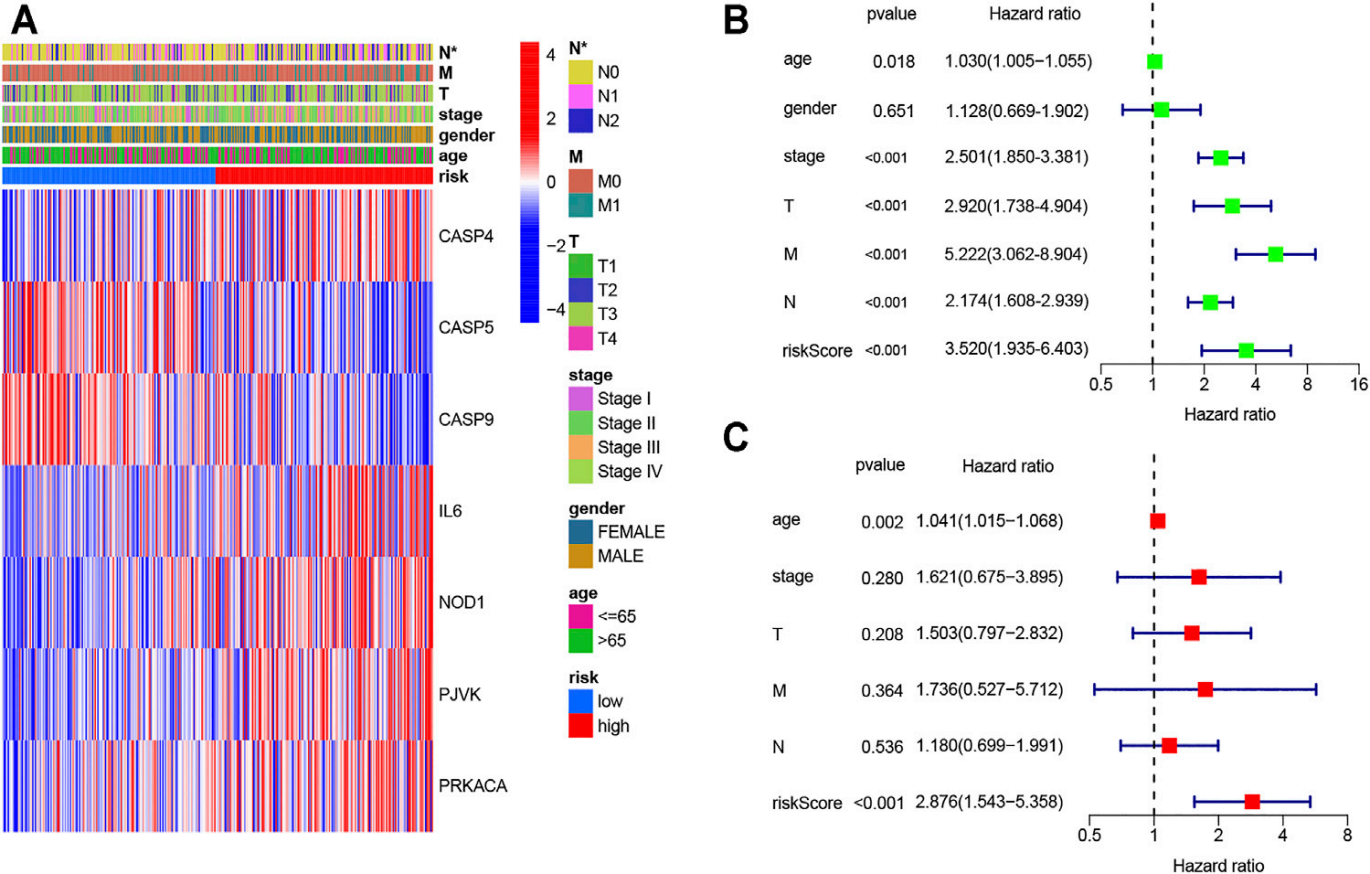
Univariate and multivariate Cox regression analyses for the risk score. **(A)** Heatmap (blue: low expression; red: high expression) for the connections between clinicopathological features and the risk groups (*p < 0.05); **(B)** univariable Cox regression analysis for the risk score; **(C)** multivariable Cox regression analysis for the risk score.

The AUC of ROC curves in 1-year, 3-year, and 5-year were 0.630, 0.685, and 0.756, respectively (Figure 4H). In TCGA database, the expression levels of seven genes in different groups of tumor patients and corresponding clinical information are shown in Figure 5A. The univariate and multivariate Cox regression were used to evaluate whether the risk model in our study was the independent prognostic factor in COAD patients. As shown in Figures 5B,C, the risk score model was the independent prognostic factor for COAD.

In order to further understand patients who were more suitable for our risk model, patients were grouped according to different clinicopathological features and then prognostic analysis was performed. As shown in Supplementary Figures S2A–F, the patients with parameters such as age < = 65, male, stage I–II, T3–4, N0, and M0 seem more suitable for our risk model. This result suggested that our model was more suitable for *in situ* invasive COAD.

### Functional Enrichment Analysis and Alteration of Seven Genes in the Model

The cBioPortal database was used to analyze the mutations of seven genes. Of the 524 patients with colorectal cancer, 247 had mutations in the genes in the seven-gene model (Supplementary Figure S3A). Of 332 colon cancer patients, 45.78% had the mutation (Supplementary Figure S3B). Functional analysis of 7 pyroptosis-related genes by GeneMANIA showed that the function of these genes focused on the inflammasome complex, positive regulation of cysteine-type endopeptidase activity, positive regulation of cysteine-type endopeptidase activity involved in the apoptotic process, and so on (Supplementary Figure S3C). These results indicated that the set of seven genes was able to greatly expand the level of difference detected in COAD patients. At the same time, the functions of the seven genes and their interactions showed that the genes in our gene model were mainly involved in inflammatory processes and cell death processes.

### Construction and Validation of the Nomogram Based on Clinicopathological Features and Risk Score

**FIGURE 6:**
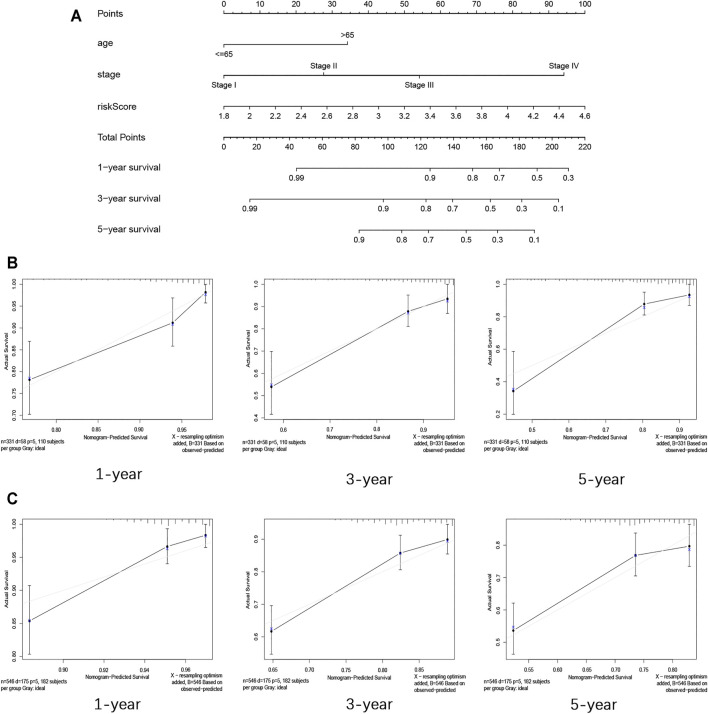
Construction and validation of the nomogram for COAD. **(A)** Nomogram for predicting the 1-year, 3-year, and 5-year survival rate by age, stage, and risk score. **(B)** 1-, 3-, and 5-year calibration curves of TCGA dataset. **(C)** 1-, 3-, and 5-year calibration curves of GEO dataset.

The nomogram in this research was established by the clinicopathological features (age and stage) and risk score based on the data from TCGA (Figure 6A). The C-index of the nomogram in predicting the survival rate was 0.774. The calibration curves for internal validation are shown in Figure 6B. The calibration curves for external validation are shown in Figure 6C. These results showed that the ability in predicting the prognosis of COAD patients can be improved by combining clinicopathological features with the risk score. At the same time, our verification also proved that this nomogram can well predict the prognosis of patients.

### Correlation of the Genes and Risk Score with Clinicopathological Features and Immune Cells as well as Immune Signaling Pathways

**FIGURE 7:**
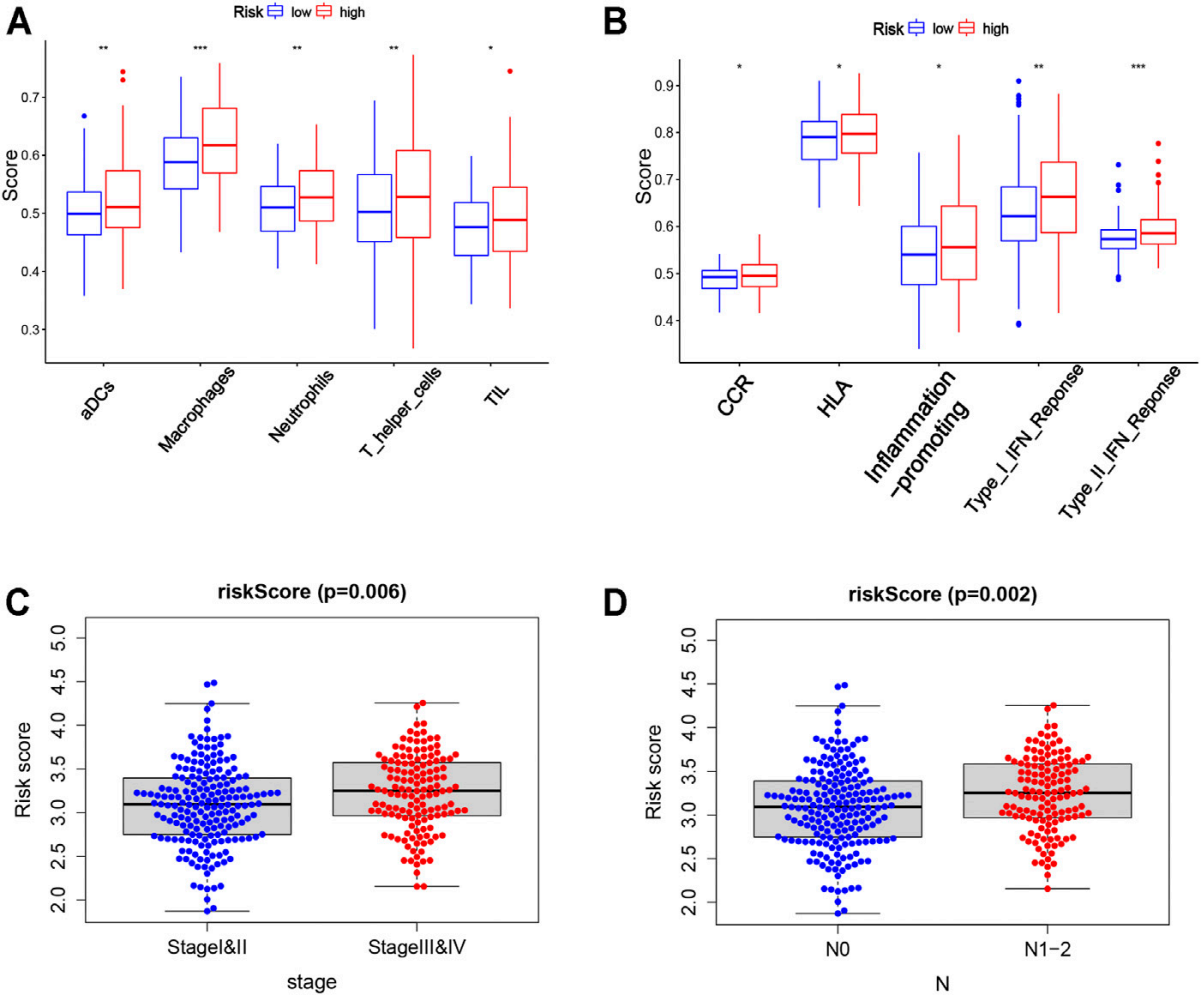
Correlation of the risk score with clinicopathological features and immune cells as well as immune function. **(A,B)** Comparison of the enrichment scores of 5 types of immune cells and 5 immune-related functions between low- (blue box) and high-risk (red box) groups in the TCGA cohort. **(C,D)** Correlation between the risk score and clinicopathological features. Stage, tumor–node–metastasis (TNM) stage; N, node.

Based on the risk score, the patients from TCGA were divided into two groups (low-risk and high-risk groups). Enrichment scores of 16 types of immune cells and the activity of 13 functional analyses of immune-related pathways were compared between two groups by the single-sample gene set enrichment analysis (ssGSEA). In TCGA cohort, the high-risk group was associated with higher levels of infiltration of activated dendritic cells (aDCs), macrophages, neutrophils, T-helper cells, and tumor infiltrating lymphocytes (TILs) (Figure 7A). In addition, the immune-related function enrichment of the high-risk group is shown in Figure 7B. Other immune cells and the immune-related function enrichment that did not show significant difference were shown in Supplementary Figures S4A–B. The risk score was greatly associated with clinicopathological factors of TCGA-COAD (*p* < 0.05; Figures 7C,D). The high-risk score was associated with the advanced TNM stage and N stage. The association between genes in risk models and clinicopathological features is shown in Supplementary Figure S5.

### The mRNA Relative Expression of Seven Genes

**FIGURE 8:**
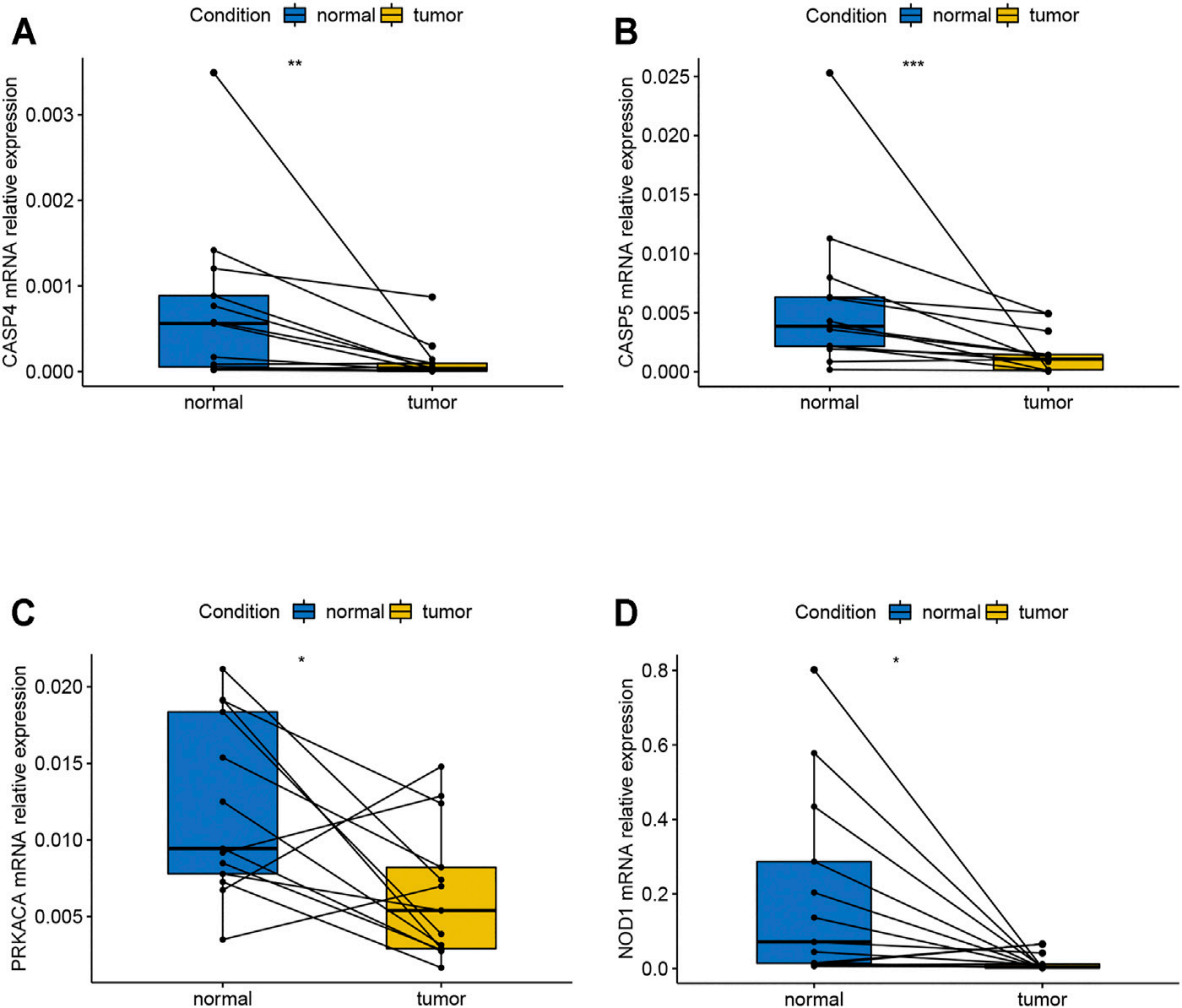
mRNA relative expression of genes in the risk model by the method of qPCR. **(A)** mRNA relative expression of CASP4; **(B)** mRNA relative expression of CASP5; **(C)** mRNA relative expression of PRCAKA; **(D)** mRNA relative expression of NOD1. qPCR, quantitative real-time polymerase chain reaction.

Based on the verification of the tissues from COAD patients by the method of qPCR, the results showed that CASP4, CASP5, PRKACA, and NOD1 were expressed differentially between normal and tumor tissues (Figures 8A–D). The corresponding *p*-values of CASP4, CASP5, PRKACA, and NOD1 were 0.0384, 0.0392, 0.0173, and 0.0288, respectively. In addition, as shown in Supplementary Figures S6A–C, the expression of CASP9, IL6, and PJVK did not show significant difference, but the trend of the gene expression between tumor and normal tissue can still be seen, which may require us to expand the sample size to further prove. In conclusion, relevant validation results suggested that most of the genes in the model have different expressions, and more samples may be required for further validation.

## Discussion

To our knowledge, this was the first study to use pyroptosis-related genes to construct a risk model to predict prognosis of patients with COAD. In this study, the results indicated that pyroptosis-related gene risk model can be used to predict the prognosis of COAD. Meanwhile, the risk model was associated with clinicopathological factors, immune cells, and immune-related functions.

Pyroptosis is associated with many diseases (Al Mamun et al., 2020; Al Mamun et al., 2021; Chu et al., 2016; Wang et al., 2018). Meanwhile, pyroptosis may mediate cell death by Caspase-1–dependent, Caspase-4/5/11–dependent, and Caspase-3/8–dependent inflammasome signaling pathways (Antonopoulos et al., 2015; Yu et al., 2019; Chen et al., 2020; Liu et al., 2021; L.; Wang et al., 2021). Recently, increasing studies show that pyroptosis is associated with the development of cancer (Al Mamun et al., 2021). Wang et al. indicted that 5-FU can induce pyroptosis in gastric cancer cell by Caspase-3 signaling pathways (Wang et al., 2018). Chu et al. showed that the signaling pathway of pyroptosis participated in the development of hepatocellular carcinoma and may become the target for treatment (Chu et al., 2016). As for COAD, Hu et al. found that pyroptosis was involved in the tumor development of colitis-related COAD (Hu et al., 2010). Chen et al. showed that pyroptosis played important roles in growth and metastasis of COAD (Tang et al., 2020). These studies suggested that pyroptosis-related genes may be the potential biomarkers for cancer. However, no studies have used pyroptosis-related genes to construct a risk model in COAD. In our study, data from TCGA were used as a training set, and data from GEO were used as a validation set to construct a seven-pyroptosis-related risk model by using univariable and LASSO Cox regression analyses. The prognostic model can well predict the prognosis of patients, which will be helpful to clinical evaluation and provide new therapeutic targets.

Genes in this risk model have been reported in studies of cancer. The Caspase 4 (CASP4) gene encodes a protein involved in immunity and inflammation (McIlwain et al., 2013). As the tumor-suppressor gene, CASP4 is associated with the poor outcome of esophageal squamous cell carcinoma (Shibamoto et al., 2017). Meanwhile, a study suggested that CASP4 may become the potential biomarker for diagnosis and treatment of tumors (Flood et al., 2015). Caspase 5 (CASP5) is an acknowledged frameshift target in the microsatellite instability gastrointestinal tract (Schwartz et al., 1999; Trojan et al., 2004). Caspase 9 (CASP9) can target colorectal cancer stem cells by inducible CASP9 to decrease the tumor size (Kemper et al., 2012). Interleukin 6 (IL6) is an important mediator of inflammatory responses and contributes to the development of inflammatory diseases (Koper-Lenkiewicz et al., 2021). IL6 plays an important role in the autophagy and chemotherapy resistance of COAD (Hu et al., 2021). Nucleotide oligomerization domain receptor 1 (NOD1) is a cytoplasmic pattern recognition receptor (Jiang et al., 2020). Some studies show that NOD1 can promote the carcinogenesis and metastasis of COAD, which may be the target to reduce postoperative recurrence (Jiang et al., 2020; Maisonneuve et al., 2021). Pejvakin (PJVK) is related to various auditory phenotypes in patients (Cheng et al., 2020). In serous ovarian cancer, the PJVK expression is downregulated, but its significance in tumors is unclear and needs further investigation (Berkel and Cacan, 2021). Protein kinase cAMP–activated catalytic subunit alpha (PRKACA) can interact with protein kinase cAMP–dependent type I regulatory subunit alpha (PRKAR1A) to inhibit the activity of protein kinase A, which may participate in the growth of COAD (Tseng et al., 2017; Zhao et al., 2021). In phosphorylation, PRKACA can help C16-ceramide to induce EMD phosphorylation so that it enhances the autophagosomal formation in COAD (Deroyer et al., 2014). These studies suggested that seven pyroptosis-related genes are associated with tumor. Our risk model was constructed by the tumor-related genes.

Survival analysis revealed that the risk model based on pyroptosis-related genes was more suitable for young males with early-stage cancer. In addition, the risk model was associated with immune cells (macrophages, neutrophils, and so on). These results indicated that our risk model was related to the tumor microenvironment.

Though our risk model can well predict the prognosis of COAD, there are still a few limitations. On the one hand, our risk model needs to collect data for further validation. On the other hand, some of the genes in the model need more samples for further validation and further study on their mechanism of action in COAD.

## Conclusion

In summary, our study revealed that pyroptosis-related genes showed great difference between normal and tumor tissues in COAD, and some of the genes in the risk model were validated in 13 patients with COAD. Moreover, the risk score based on seven pyroptosis-related genes was the independent factors for COAD and can well predict the prognosis. In addition, our model was more suitable for the early-stage patients, which may be regarded as the method to perform early diagnosis for tumor patients. Therefore, we thought our study can help identify patients in the early stages and may provide potentially effective new targets for the treatment of cancer patients.

## Data Availability

The datasets presented in this study can be found in online repositories. The names of the repository/repositories and accession number(s) can be found in the article/Supplementary Material.

## References

[B1] Al Mamun A. Mimi A. A. Aziz M. A. Zaeem M. Ahmed T. Munir F. (2021). Role of Pyroptosis in Cancer and its Therapeutic Regulation. Eur. J. Pharmacol. 910, 174444. 10.1016/j.ejphar.2021.174444 34453928

[B2] Al Mamun A. Mimi A. A. Zaeem M. Wu Y. Monalisa I. Akter A. (2021). Role of Pyroptosis in Diabetic Retinopathy and its Therapeutic Implications. Eur. J. Pharmacol. 904, 174166. 10.1016/j.ejphar.2021.174166 33979651

[B3] Al Mamun A. Wu Y. Jia C. Munir F. Sathy K. J. Sarker T. (2020). Role of Pyroptosis in Liver Diseases. Int. Immunopharmacology 84, 106489. 10.1016/j.intimp.2020.106489 32304992

[B4] Antonopoulos C. Russo H. M. El Sanadi C. Martin B. N. Li X. Kaiser W. J. (2015). Caspase-8 as an Effector and Regulator of NLRP3 Inflammasome Signaling. J. Biol. Chem. 290 (33), 20167–20184. 10.1074/jbc.M115.652321 26100631 PMC4536427

[B5] Berkel C. Cacan E. (2021). Differential Expression and Copy Number Variation of Gasdermin (GSDM) Family Members, Pore-Forming Proteins in Pyroptosis, in Normal and Malignant Serous Ovarian Tissue. Inflammation. 10.1007/s10753-021-01493-0 34091823

[B6] Bray F. Ferlay J. Soerjomataram I. Siegel R. L. Torre L. A. Jemal A. (2018). Global Cancer Statistics 2018: GLOBOCAN Estimates of Incidence and Mortality Worldwide for 36 Cancers in 185 Countries. CA: A Cancer J. Clinicians 68 (6), 394–424. 10.3322/caac.21492 30207593

[B7] Broz P. Pelegrín P. Shao F. (2020). The Gasdermins, a Protein Family Executing Cell Death and Inflammation. Nat. Rev. Immunol. 20 (3), 143–157. 10.1038/s41577-019-0228-2 31690840

[B8] Chen K. W. Demarco B. Broz P. (2020). Beyond Inflammasomes: Emerging Function of Gasdermins during Apoptosis and NETosis. EMBO J. 39 (2), e103397. 10.15252/embj.2019103397 31793683 PMC6960442

[B9] Cheng Y.-F. Tsai Y.-H. Huang C.-Y. Lee Y.-S. Chang P.-C. Lu Y.-C. (2020). Generation and Pathological Characterization of a Transgenic Mouse Model Carrying a Missense PJVK Mutation. Biochem. Biophysical Res. Commun. 532 (4), 675–681. 10.1016/j.bbrc.2020.07.101 32917362

[B10] Chu Q. Jiang Y. Zhang W. Xu C. Du W. Tuguzbaeva G. (2016). Pyroptosis Is Involved in the Pathogenesis of Human Hepatocellular Carcinoma. Oncotarget 7 (51), 84658–84665. 10.18632/oncotarget.12384 27705930 PMC5356689

[B11] Deroyer C. Rénert A.-F. Merville M.-P. Fillet M. (2014). New Role for EMD (Emerin), a Key Inner Nuclear Membrane Protein, as an Enhancer of Autophagosome Formation in the C16-Ceramide Autophagy Pathway. Autophagy 10 (7), 1229–1240. 10.4161/auto.28777 24819607 PMC4203549

[B12] Fink S. L. Cookson B. T. (2006). Caspase-1-dependent Pore Formation during Pyroptosis Leads to Osmotic Lysis of Infected Host Macrophages. Cell Microbiol 8 (11), 1812–1825. 10.1111/j.1462-5822.2006.00751.x 16824040

[B13] Fleming M. Ravula S. Tatishchev S. F. Wang H. L. (2012). Colorectal Carcinoma: Pathologic Aspects. J. Gastrointest. Oncol. 3 (3), 153–173. 10.3978/j.issn.2078-6891.2012.030 22943008 PMC3418538

[B14] Flood B. Oficjalska K. Laukens D. Fay J. O'Grady A. Caiazza F. (2015). Altered Expression of Caspases‐4 and ‐5 during Inflammatory Bowel Disease and Colorectal Cancer: Diagnostic and Therapeutic Potential. Clin. Exp. Immunol. 181 (1), 39–50. 10.1111/cei.12617 25943872 PMC4469154

[B15] Guo J. Zheng J. Mu M. Chen Z. Xu Z. Zhao C. (2021). GW4064 Enhances the Chemosensitivity of Colorectal Cancer to Oxaliplatin by Inducing Pyroptosis. Biochem. Biophysical Res. Commun. 548, 60–66. 10.1016/j.bbrc.2021.02.043 33631675

[B16] Hu B. Elinav E. Huber S. Booth C. J. Strowig T. Jin C. (2010). Inflammation-induced Tumorigenesis in the colon Is Regulated by Caspase-1 and NLRC4. Proc. Natl. Acad. Sci. 107 (50), 21635–21640. 10.1073/pnas.1016814108 21118981 PMC3003083

[B17] Hu F. Song D. Yan Y. Huang C. Shen C. Lan J. (2021). IL-6 Regulates Autophagy and Chemotherapy Resistance by Promoting BECN1 Phosphorylation. Nat. Commun. 12 (1), 3651. 10.1038/s41467-021-23923-1 34131122 PMC8206314

[B18] Jiang H. Y. Najmeh S. Martel G. MacFadden-Murphy E. Farias R. Savage P. (2020). Activation of the Pattern Recognition Receptor NOD1 Augments colon Cancer Metastasis. Protein Cell 11 (3), 187–201. 10.1007/s13238-019-00687-5 31956962 PMC7026222

[B19] Kemper K. Rodermond H. Colak S. Grandela C. Medema J. P. (2012). Targeting Colorectal Cancer Stem Cells with Inducible Caspase-9. Apoptosis 17 (5), 528–537. 10.1007/s10495-011-0692-z 22223359 PMC3322325

[B20] Koncina E. Haan S. Rauh S. Letellier E. (2020). Prognostic and Predictive Molecular Biomarkers for Colorectal Cancer: Updates and Challenges. Cancers 12 (2), 319. 10.3390/cancers12020319 PMC7072488 32019056

[B21] Koper-Lenkiewicz O. M. Dymicka-Piekarska V. Milewska A. J. Zińczuk J. Kamińska J. (2021). The Relationship between Inflammation Markers (CRP, IL-6, sCD40L) and Colorectal Cancer Stage, Grade, Size and Location. Diagnostics 11 (8), 1382. 10.3390/diagnostics11081382 34441316 PMC8393680

[B22] Liu B. He R. Zhang L. Hao B. Jiang W. Wang W. (2021). Inflammatory Caspases Drive Pyroptosis in Acute Lung Injury. Front. Pharmacol. 12, 631256. 10.3389/fphar.2021.631256 33613295 PMC7892432

[B23] Loveless R. Bloomquist R. Teng Y. (2021). Pyroptosis at the Forefront of Anticancer Immunity. J. Exp. Clin. Cancer Res. 40 (1), 264. 10.1186/s13046-021-02065-8 34429144 PMC8383365

[B24] Maisonneuve C. Tsang D. K. L. Foerster E. G. Robert L. M. Mukherjee T. Prescott D. (2021). Nod1 Promotes Colorectal Carcinogenesis by Regulating the Immunosuppressive Functions of Tumor-Infiltrating Myeloid Cells. Cel Rep. 34 (4), 108677. 10.1016/j.celrep.2020.108677 33503439

[B25] McIlwain D. R. Berger T. Mak T. W. (2013). Caspase Functions in Cell Death and Disease. Cold Spring Harbor Perspect. Biol. 5 (4), a008656. 10.1101/cshperspect.a008656 PMC3683896 23545416

[B26] Schwartz S. Jr. Yamamoto H. Navarro M. Maestro M. Reventós J. Perucho M. (1999). Frameshift Mutations at Mononucleotide Repeats in Caspase-5 and Other Target Genes in Endometrial and Gastrointestinal Cancer of the Microsatellite Mutator Phenotype. Cancer Res. 59 (12), 2995–3002. 10383166

[B27] Sepulveda A. R. Hamilton S. R. Allegra C. J. Grody W. Cushman-Vokoun A. M. Funkhouser W. K. (2017). Molecular Biomarkers for the Evaluation of Colorectal Cancer. J. Mol. Diagn. 19 (2), 187–225. 10.1016/j.jmoldx.2016.11.001 28185757 PMC5971222

[B28] Shibamoto M. Hirata H. Eguchi H. Sawada G. Sakai N. Kajiyama Y. (2017). The Loss of CASP4 Expression Is Associated with Poor Prognosis in Esophageal Squamous Cell Carcinoma. Oncol. Lett. 13 (3), 1761–1766. 10.3892/ol.2017.5646 28454321 PMC5403698

[B29] Tan G. Huang C. Chen J. Zhi F. (2020). HMGB1 Released from GSDME-Mediated Pyroptotic Epithelial Cells Participates in the Tumorigenesis of Colitis-Associated Colorectal Cancer through the ERK1/2 Pathway. J. Hematol. Oncol. 13 (1), 149. 10.1186/s13045-020-00985-0 33160389 PMC7648939

[B30] Tang Z. Ji L. Han M. Xie J. Zhong F. Zhang X. (2020). Pyroptosis Is Involved in the Inhibitory Effect of FL118 on Growth and Metastasis in Colorectal Cancer. Life Sci. 257, 118065. 10.1016/j.lfs.2020.118065 32659366

[B31] Trojan J. Brieger A. Raedle J. Weber N. Kriener S. Kronenberger B. (2004). BAX and Caspase-5 Frameshift Mutations and Spontaneous Apoptosis in Colorectal Cancer with Microsatellite Instability. Int. J. Colorectal Dis. 19 (6), 538–544. 10.1007/s00384-004-0597-1 15088110

[B32] Tseng I.-C. Huang W.-J. Jhuang Y.-L. Chang Y.-Y. Hsu H.-P. Jeng Y.-M. (2017). Microinsertions inPRKACAcause Activation of the Protein Kinase A Pathway in Cardiac Myxoma. J. Pathol. 242 (2), 134–139. 10.1002/path.4899 28369983

[B33] Wang L. Qin X. Liang J. Ge P. (2021). Induction of Pyroptosis: A Promising Strategy for Cancer Treatment. Front. Oncol. 11, 635774. 10.3389/fonc.2021.635774 33718226 PMC7953901

[B34] Wang Y. Gao W. Shi X. Ding J. Liu W. He H. (2017). Chemotherapy Drugs Induce Pyroptosis through Caspase-3 Cleavage of a Gasdermin. Nature 547 (7661), 99–103. 10.1038/nature22393 28459430

[B35] Wang Y. Yin B. Li D. Wang G. Han X. Sun X. (2018). GSDME Mediates Caspase-3-dependent Pyroptosis in Gastric Cancer. Biochem. Biophysical Res. Commun. 495 (1), 1418–1425. 10.1016/j.bbrc.2017.11.156 29183726

[B36] Warde-Farley D. Donaldson S. L. Comes O. Zuberi K. Badrawi R. Chao P. (2010). The GeneMANIA Prediction Server: Biological Network Integration for Gene Prioritization and Predicting Gene Function. Nucleic Acids Res. 38, W214–W220. Web Server issue. 10.1093/nar/gkq537 20576703 PMC2896186

[B37] Ye Y. Dai Q. Qi H. (2021). A Novel Defined Pyroptosis-Related Gene Signature for Predicting the Prognosis of Ovarian Cancer. Cell Death Discov. 7 (1), 71. 10.1038/s41420-021-00451-x 33828074 PMC8026591

[B38] Yu J. Li S. Qi J. Chen Z. Wu Y. Guo J. (2019). Cleavage of GSDME by Caspase-3 Determines Lobaplatin-Induced Pyroptosis in colon Cancer Cells. Cell Death Dis 10 (3), 193. 10.1038/s41419-019-1441-4 30804337 PMC6389936

[B39] Zhai X. Xue Q. Liu Q. Guo Y. Chen Z. (2017). Colon Cancer Recurrence-Associated Genes Revealed by WGCNA Co-expression Network Analysis. Mol. Med. Rep. 16 (5), 6499–6505. 10.3892/mmr.2017.7412 28901407 PMC5865817

[B40] Zhao Y. Wang Y. Zhao J. Zhang Z. Jin M. Zhou F. (2021). PDE2 Inhibits PKA-Mediated Phosphorylation of TFAM to Promote Mitochondrial Ca2+-Induced Colorectal Cancer Growth. Front. Oncol. 11, 663778. 10.3389/fonc.2021.663778 34235078 PMC8256694

